# Diagnostic Yield of Xpert MTB/RIF Assay Using Bronchoalveolar Lavage Fluid in Detecting *Mycobacterium tuberculosis* among the Sputum-Scarce Suspected Pulmonary TB Patients

**DOI:** 10.3390/diagnostics12071676

**Published:** 2022-07-10

**Authors:** Mohammad Khaja Mafij Uddin, Md. Fahim Ather, Sharmin Akter, Rumana Nasrin, Tanjina Rahman, Sk Nazmul Kabir, S. M. Mazidur Rahman, Stephane Pouzol, Jonathan Hoffmann, Sayera Banu

**Affiliations:** 1 Infectious Diseases Division, International Centre for Diarrhoeal Disease Research, Dhaka 1212, Bangladesh; kmuddin@icddrb.org (M.K.M.U.); fahim.ather@icddrb.org (M.F.A.); ahmedsetu61@gmail.com (S.A.); rumana.nasrin@icddrb.org (R.N.); tanjina.rahman@icddrb.org (T.R.); nazmul.kabir@icddrb.org (S.N.K.); smmazidur@icddrb.org (S.M.M.R.); 2 Scientific and Medical Department, Fondation Mérieux, 17 Rue Bourgelat, 69002 Lyon, France; stephane.pouzol@fondation-merieux.org (S.P.); jonathan.hoffmann@fondation-merieux.org (J.H.)

**Keywords:** pulmonary TB, *Mycobacterium tuberculosis*, bronchoalveolar lavage fluid, Xpert MTB/RIF assay

## Abstract

Tuberculosis (TB) remains one of the leading causes of death worldwide and is caused by the single infectious agent *Mycobacterium tuberculosis* (Mtb). Although sputum is the most common specimen for pulmonary TB detection, some other respiratory specimens, such as bronchoalveolar lavage (BAL) fluid, gastric lavage (GL), and induced sputum (IS), are also collected from patients who are unable to deliver sputum. In this study, we aimed to evaluate the diagnostic performances of different test methods for TB diagnosis using BAL fluid specimens from sputum-scarce pulmonary TB patients. In this current study, a total of 210 BAL fluid specimens were collected and subjected to culture on Lowenstein–Jensen (L-J) medium, using an N-acetyl-L-cysteine-Sodium Hydroxide decontamination and digestion method, Xpert MTB/RIF (Xpert, Cepheid, Sunnyvale, CA, USA) assay, and acid-fast bacilli (AFB) microscopy with a Ziehl–Neelsen staining method for the detection of pulmonary TB. The sensitivity and specificity of these methods were then analyzed against the composite reference standard (CRS). Additionally, the receiver operating characteristic (ROC) curve was used to evaluate the diagnostic value of these assays. Among the 210 specimens, 39 (18.6%), 27 (12.8%), and 12 (5.7%) were found positive with Xpert assay, culture, and AFB microscopy, respectively. Considering the CRS, 42 (20%) were positive as the final diagnosis. The Xpert assay had a significantly higher sensitivity (92.9%, 95% CI: 80.5–98.5) compared to culture (64.3%, 95% CI: 48.0–78.4) and AFB microscopy (28.6%, 95% CI: 15.7–44.6) against the CRS. Additionally, the area under the ROC curve (AUC) for the Xpert assay, culture, and AFB microscopy accounted for 0.964, 0.821, and 0.655, respectively, when using CRS as the reference. In conclusion, our study findings demonstrated that the Xpert assay conferred a considerable diagnostic potential compared to other conventional methods for the diagnosis of pulmonary TB from BAL fluid specimens.

## 1. Introduction

Tuberculosis (TB) is one of the leading communicable diseases globally and occurs due to infection by *Mycobacterium tuberculosis* (Mtb) bacillus. Until the Coronavirus disease (COVID-19) pandemic, TB remained the leading cause of death worldwide from a single infectious agent (ranking above HIV/AIDS). According to the Global TB Report 2021, an estimated 9.9 million people were infected with TB, where newly diagnosed TB cases fell from 7.1 million in 2019 to 5.8 million in 2020 [1]. This resulted from the reduced access to TB diagnosis and treatment due to the COVID-19 pandemic and eventually increased the TB deaths to an estimated 1.3 million in 2020 (around 1.2 million in 2019). Bangladesh remains one of the 16 countries with a burden of high TB and multi-drug resistant TB (MDR-TB). According to an annual report in 2021, approximately 360 thousand people were TB infected with around 44,000 deaths, where laboratory confirmed MDR and Pre-XDR/XDR-TB cases were 1113 and 67, respectively, in Bangladesh [1,2,3].

Early diagnosis of active TB is essential for disease management, both for treating the infected cases and for reducing the risk of transmission in the community [4]. In clinical practice, rapid TB diagnosis continues to be a considerable challenge for clinicians. The conventional methods available for diagnosis are acid-fast bacilli (AFB) smear microscopy and mycobacterial culture. AFB microscopy is a widely used, rapid, and reasonable method for TB detection, but it has poor sensitivity, especially for the paucibacillary specimens, and a high proportion (20–66%) of TB cases are smear-negative [5,6]. In addition to these, poor quality of sputum and microscopic observation can also contribute to smear-negative results, which ultimately reduce the sensitivity of AFB smears in diagnosis of TB [7]. On the other hand, despite being considered the gold standard for TB detection, culture does not provide a prompt result and requires 2–8 weeks for final determination. Additionally, laboratory expertise and sophisticated biosafety facilities are required to conduct culture testing [8,9,10]. Therefore, advancement and innovative developments in molecular diagnostics for TB have been introduced, playing a crucial role in rapid detection and response, for better TB control at the global level [11,12]. During the last two decades, several molecular methods have been introduced for TB diagnosis along with determination of the type and extent of drug resistance of Mtb. GenoType MTBDR*plus* (Line Probe Assay; Hain Life Sciences, Nehren, Germany), Xpert MTB/RIF (Xpert), and an improved version of Xpert MTB/RIF Ultra assay (Cepheid, Sunnyvale, CA, USA) are such methods, which can simultaneously detect TB and determine the type and extent of drug resistance in both respiratory and non-respiratory specimens. The Xpert assay is a World Health Organization (WHO)-recommended hemi-nested real-time polymerase chain reaction technology that detects *M*. *tuberculosis* complex (MTBC) as well as rifampicin (RIF) resistance within two hours [9,13,14]. This automated technology not only provides prompt TB detection but also detects very low genomic copies of MTBC in various clinical specimens [8,15].

Occasionally, suspected cases of TB (with radiographic evidence) are unable to expectorate sputum; thus clinical specimens alternative to sputum are required for TB diagnosis. In such cases, bronchoalveolar lavage (BAL) fluid, gastric lavage (GL), and induced sputum (IS) are the clinical specimens that are used. Subsequently, some authors reported that BAL fluid is a more suitable sample than GL or IS for the diagnosis of pulmonary TB in this subgroup of population [16,17]. In this study, we evaluated the diagnostic performances of different test methods for TB diagnosis using BAL fluid specimens among sputum-scarce suspected pulmonary TB patients.

## 2. Materials and Methods

### 2.1. Specimen Collection

**Figure 1:**
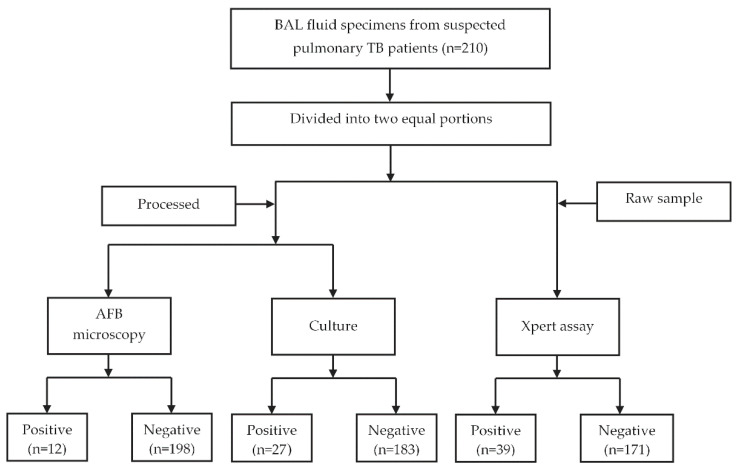
Study flowchart explaining the patient enrolment, methodology, and test results.

Study participants were enrolled from the outpatient services of Mohakhali TB Screening and Treatment Center, Dhaka, Bangladesh. A total of 210 patients with symptoms of chest radiography or suggestive of pulmonary TB were enrolled in this study in the period of January 2019 to December 2019. According to the inclusion criteria, suspected patients (who were unable to produce or expectorate sputum) were referred to the pulmonologists for the collection of BAL fluid (minimum 5.0 mL of volume) for clinical investigations. Inability to provide an adequate amount of BAL fluid specimen was considered an exclusion criterion [18]. A brief study questionnaire was used to collect the demographic and clinical history. All of the laboratory tests were performed at the Mycobacteriology Laboratory of icddr,b. Each specimen was divided into two equal portions: one portion was used for Xpert assay, and the other portion was used for culture and AFB microscopy. The overall study flowchart is shown in Figure 1.

### 2.2. Specimen Processing

All of the specimens were processed according to the NALC-NaOH (N-acetyl-L-cysteine-Sodium Hydroxide) decontamination and digestion method [19]. Briefly, equal volumes of BAL fluid and NALC-NaOH-Na Citrate solution (0.5% NALC and 4% NaOH-2.94% Na-Citrate) were added, mixed by vortexing at least for 20 s, and then incubated at room temperature for 15 min. The tubes were then shaken by hand for 5 and 10 min during the incubation and neutralized with phosphate buffer saline (PBS, pH = 6.8). The mixture was centrifuged at 3000× *g* for 15 min, and the supernatant was decanted carefully. The pellet was then resuspended with 1.0 mL of PBS and used for culture and AFB microscopy.

### 2.3. Culture and AFB Microscopy

Each processed specimen was inoculated on two solid Lowenstein–Jensen (L–J) slants and incubated at 37 °C for up to 8 weeks. Within this period, the media were examined weekly for visible bacterial colonies, and mycobacterial growth was confirmed by a specific polymerase chain reaction (PCR) [20]. No visible growth on either L-J slant was categorized as culture negative. Processed specimens were also subjected to AFB microscopy, as described earlier [6].

### 2.4. Insertion Sequence 6110 (IS6110) PCR

To confirm the growth of MTBC on L-J slants, IS*6110* PCR was performed. Genomic DNA (Deoxyribonucleic acid) was extracted from the fresh culture of each specimen by following the standard protocol described previously [21]. The extracted DNA samples were used for PCR amplification using the IS*6110* primer set: IS6110 F (5′-CCTGCGAGCGTAGGCGTCGG-3′) and IS6110 R (5′-CTCGTCCAGCGCCGCTTCGG-3′), and the amplified 123 bp PCR products confirmed the presence of MTBC in the culture [22]. In every PCR run, a positive control (DNA from H37Rv) and a negative control (nuclease-free water instead of template) were used.

### 2.5. Xpert MTB/RIF Assay

The other portion (raw part) of each BAL fluid specimen was subjected to Xpert assay, according to the manufacturer’s instructions [15]. In brief, sample reagent was added to unprocessed BAL fluid specimen in a 2:1 ratio to a 15 mL microcentrifuge tube and mixed by vortexing. The mixture was then incubated at room temperature for 15 min and gently vortexed once in the middle of this period. Then, 2.0 mL of the liquefied specimen was transferred into an Xpert test cartridge (version 5.0) and loaded onto the Xpert machine. The auto-generated results were recorded from the Xpert software (version 4.8).

### 2.6. Data Analysis

All the collected data were entered into Statistical Package for the Social Sciences software (SPSS) version 20.0. Statistical analysis was performed using STATA version 17.0. The sensitivity, specificity, and predictive values were calculated with 95% confidence intervals (CIs). McNemar’s test was used for the comparison of sensitivities, where *p* < 0.05 was considered statistically significant. The overall performances of the tested diagnostic methods were evaluated using the area under the receiver operating characteristic (ROC) curve (AUC) using RStudio version 1.4. An AUC with >0.5 to <0.7 indicated a low diagnostic value, ≥0.7 to <0.9 indicated a moderate diagnostic value, and ≥0.9 indicated a high diagnostic value [23]. The composite reference standard (CRS) of this study consisted of the AFB microscopy, culture, or Xpert assay that determined the final diagnosis of TB [24].

## 3. Results

### 3.1. Demographic Characteristics and Clinical Presentations of the Enrolled Patients

The demographic and clinical characteristics for all of the 210 patients are shown in Table 1. Among the 210 enrolled participants, 140 (66.7%) were male, and the median age of the patients was 49 years. The majority of the patients were comparatively of an older age group (59% were ≥45 years of age), 196 (93.3%) patients had no previous history of TB, and 85.2% of the enrolled participants were non-diabetic. According to occupation, both service holders and housewives were 20.5%, while businessmen and students were 8.5% and 8.1%, respectively (Table 1).

### 3.2. Diagnostic Performances of Different Tests Methods

Among the 210 BAL fluid specimens, a total of 39 (18.6%) were found to be positive using an Xpert assay, and all of the Xpert positive cases were identified as RIF sensitive. Depending on the bacterial load in the specimen, these Xpert assay results were grouped into high, medium, low, and very low categories. Among the 39 Xpert assay positive cases, 1, 9, 12, and 17 had high, medium, low, and very low bacterial load, respectively. Again, out of the 210 BAL fluid specimens, 27 (12.8%) were found to be positive in culture and 12 (5.7%) were positive in AFB microscopy (Table 2). Out of the 12 AFB microscopy positive cases, four were 1+, one was 2+, and the remaining seven were graded as scanty positive (ranging from scanty 5 to 8).

### 3.3. Comparative Analysis of Different Test Methods

**Figure 2:**
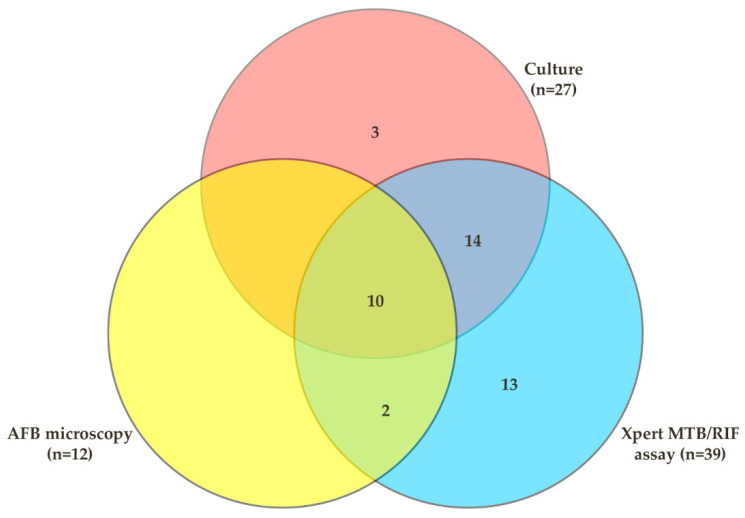
Venn diagram representing the positive diagnostic results of different test methods.

The diagnostic performance of the Xpert assay and AFB microscopy was evaluated by considering culture as the gold standard for TB diagnosis. Out of the 27 culture-positive cases, 24 were positive in the Xpert assay; the sensitivity of the Xpert assay was 88.9% (95% CI: 70.8–97.6) and the specificity was 91.8% (95% CI 86.8–95.3). However, among the culture-positive cases, 10 were positive by AFB microscopy; hence, the sensitivity and specificity of AFB microscopy were 37.0% (95% CI: 19.40–57.6) and 98.9% (95% CI: 96.1–99.8), respectively. Considering culture as the gold standard, the positive predictive values (PPV) and negative predictive values (NPV) for Xpert assay and AFB microscopy were 61.5% (95% CI: 44.6–76.6) vs. 98.2% (95% CI: 95.06–99.6) and 83.3 (95% CI: 51.6–97.9) vs. 91.4 (95% CI: 86.6–94.9), respectively (Table 3, Figure 2).

On the other hand, compared to CRS (*n* = 42); a total of 39, 27, and 12 specimens were positive with Xpert assay, culture, and AFB microscopy, respectively. Considering CRS as the final diagnosis, Xpert assay had a significantly higher sensitivity of (92.9%, 95% CI: 80.5–98.5, *p* = 0.0047) compared to culture (64.3%, 95% CI: 48.0–78.4) and AFB microscopy (28.6%, 95% CI: 15.7–44.6, *p* < 0.001). The NPV for Xpert assay, culture, and AFB microscopy were 98.2% (95% CI: 95.0–99.6), 91.8% (95% CI: 86.8–95.3), and 84.8% (95% CI: 79.1–89.5), respectively, against CRS (Table 3).

**Figure 3:**
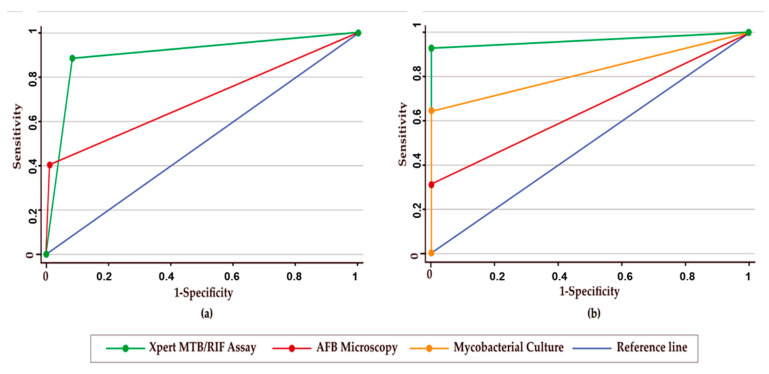
ROC curve analysis of different test methods. (**a**) The AUC of different diagnostic methods when compared to culture; (**b**) the AUC of different diagnostic methods when compared to CRS as the case group.

Additionally, the ROC curve was used to evaluate the diagnostic performances of Xpert assay, culture, and AFB microscopy. Considering culture as the reference, the AUC of Xpert assay and AFB microscopy were 0.904 and 0.698, respectively. On the other hand, the AUC of the Xpert assay, culture, and AFB microscopy accounted for 0.964, 0.821, and 0.655, respectively, when the CRS was considered as the reference (Table 4, Figure 3).

## 4. Discussion

The lack of sputum production in pulmonary TB patients is considered to be one of the major causes of negative TB diagnosis and treatment delay. Therefore, other pulmonary specimens (e.g., BAL, GL, and IS) are considered as an alternative approach for the diagnosis of such cases [25]. A number of studies showed that BAL fluid had advantages in the diagnosis of pulmonary TB in different smear-negative or sputum-scarce patients. A study conducted among HIV patients by Worodria et al. revealed that BAL fluid increased the sensitivity of TB diagnosis, from 46% to 67%, in comparison with sputum specimens [26]. Another study conducted by Menon et al. showed that BAL fluid was better than the GL, detecting 30.8% TB positive cases compared to 21.25%, respectively, in children with probable pulmonary TB [27]. However, the performance of different diagnostic methods for pulmonary TB with specimens other than sputum has not yet been reported in Bangladesh. In this study, we aimed to investigate the performance of different tests for diagnosis of TB using BAL fluid specimens. In this study, among the 210 collected BAL fluid specimens, 39 (18.6%), 27 (12.8%), and 12 (5.7%) were found to be positive in Xpert assay, culture, and AFB microscopy, respectively. According to CRS, 42 (20%) were positive and considered the final diagnosis. The sensitivity and specificity of these assays were calculated against CRS. Compared to CRS, the sensitivity of Xpert, culture, and AFB microscopy was 92.9% (95% CI: 185 80.5–98.5), 64.3% (95% CI: 48.0–78.4), and 28.6% (95% CI: 15.7–44.6), respectively.

In comparison with the culture method, Xpert assay a showed significantly higher sensitivity than AFB microscopy (88.9% vs. 37.0%, *p* < 0.001) with a diagnostic AUC of 0.904 and 0.698, respectively. The sensitivity of Xpert assay against culture was also higher in several previously published studies. Two studies, conducted by Dewald et al. and Kanwal et al., reported that the sensitivities of Xpert assay against culture for BAL fluid specimens were 92.3% and 91.86%, respectively, which were very similar to our study findings [28,29]. Conversely, the specificity of the Xpert assay was lower than AFB microscopy (91.8% vs. 98.9%) in our investigation. Similar specificities with the Xpert assay were also reported in studies conducted by Pierre et al. and Lee et al. with 98.6% and 100%, respectively, when compared to culture [30,31]. The specificity for Xpert assay against culture was lower due to the higher number of Xpert positive cases (15 cases were positive in Xpert assay but negative in culture). The PPV and NPV for Xpert assay against culture were 61.5% and 98.2%, respectively; whereas, the PPV and NPV for AFB microscopy were 83.3% and 91.4%, respectively. The PPV and NPV for Xpert assay against culture were comparable to a study conducted by Agrawal et al., where the PPV and NPV were 73.3% and 95.7%, respectively [8]. Again, considering CRS as the final diagnosis, our study found that the sensitivity of Xpert assay, culture, and AFB microscopy were 92.9%, 64.3%, and 28.6%, respectively. Our study revealed a higher sensitivity of Xpert assay (92.9%) compared to culture and AFB microscopy. The study conducted by Gowda et al. also found a higher sensitivity of Xpert assay (46.2%) compared to culture and AFB microscopy (32.0% and 11.5% respectively), while considering CRS [32]; however, the sensitivity of the Xpert assay of their study was lower than in our current study. Additionally, in this study, the diagnostic AUC for Xpert assay, culture, and AFB microscopy was 0.964, 0.821, and 0.655, respectively, when compared with CRS. The higher AUC value of the Xpert assay (0.964) demonstrated the true diagnostic potential of the assay for pulmonary TB diagnosis from BALF specimens, where culture and AFB microscopy demonstrated moderate and low diagnostic values, respectively.

In this study, three cases tested culture positive and Xpert assay negative. This discrepancy could be due to the presence of PCR inhibitors in the specimens or very low numbers of bacilli, leading to a lack of DNA for amplification in the Xpert assay. Again, culture can detect as few as 10 colony forming units (CFU)/mL of bacilli in specimens; whereas, Xpert assay demonstrates a limit of detection of 131 CFU/mL [33,34]. Moreover, these BAL fluid specimens were stained with blood, which may have affected the detection process of the Xpert assay. Similar findings were reported in another study, where an Xpert assay showed lower sensitivity for detection of TB among blood-stained sputum specimens (28%), compared to salivary sputum (66%) [35].

On the other hand, 15 specimens were found to be positive in the Xpert assay but were culture negative. This discrepancy might occur due to the principle and experimental procedure of the test methods. The Xpert assay detected DNA from both live and dead bacilli, but the culture method recovered only viable bacterial growth. Among these 15 cases, most of the participants had been taking anti-TB treatment for at least 14 days prior to the enrollment, which may have reduced the frequency of positivity in culture. A study conducted by Walters et al. reported a similar finding, where Xpert assay provided only a 14% additional yield from a BAL fluid specimen, compared to culture, where the median duration of TB treatment before bronchoscopy was 8 days [36].

There were some limitations of the study. First, the bacterial culture was performed on L-J slants rather than an automated liquid culture medium (Mycobacteria Growth Indicator Tube, MGIT 960), which is considered superior to L-J (solid medium), in response to its higher sensitivity and shorter duration for detection of Mtb [37]. Second, we did not evaluate the sensitivity and specificity of RIF resistance detection using an Xpert assay, and thus were unable to compare the genotypic pattern of drug resistance of Mtb with the phenotypic drug susceptibility.

## 5. Conclusions

The study findings revealed that Xpert assay, culture, and AFB microscopy conferred similar specificities, but the sensitivity of the Xpert assay from BAL fluid specimens was significantly higher than that of culture and AFB microscopy. Xpert assay demonstrated potential diagnostic value for rapid detection of pulmonary TB from BAL fluids among the sputum-scarce, suspected TB patients. Care should be taken regarding Xpert assay positive but culture negative cases, and the clinical history of the patients should be considered prior to the use of Xpert assay for TB diagnosis. Our study findings may thus contribute to formulating national guidelines and also help clinicians to manage these subgroups of patients. In conclusion, the study findings suggest that the Xpert assay should be considered as a first-line test for the diagnosis of pulmonary TB among these subgroups of the population.

## Figures and Tables

**Table 1:** Demographic and clinical characteristics of the enrolled participants (*n* = 210).

Categories		Numbers (*n*)	Frequency (%)
Sex	Male	140	66.7
	Female	70	33.3
Age Ranges	1–20	10	4.7
	21–50	109	52
	>50	91	43.3
TB History	Yes	14	6.7
	No	196	93.3
Diabetes	Yes	31	14.8
	No	179	85.2
Occupations	Service holders	43	20.5
	Business	18	8.5
	Housewife	43	20.5
	Student	17	8.1
	Unemployed	20	9.5
	Others *	69	32.9

* Others include rickshaw puller, garment worker, farmer, day laborer, and work on abroad.

**Table 2:** Performance of Xpert assay, AFB microscopy, and culture methods for the detection of pulmonary TB from BAL fluid specimens.

Test Methods	Xpert Assay (*n* = 210)	AFB Microscopy (*n* = 210)		Culture (*n* = 210)	
		Positive	Negative	Positive	Negative
Test Results	Detected High-1	1	0	1	0
	Detected Medium-9	5	4	8	1
	Detected Low-12	1	11	6	6
	Detected Very Low-17	5	12	9	8
	Not Detected-171	0	171	3	168
Frequency to Positivity	18.6%	5.7%		12.8%	

**Table 3:** Comparison of sensitivity, specificity and predictive values of different test methods according to culture and CRS.

Variables (95% CI)	Compared to Culture (*n* = 27)		Compared to CRS (*n* = 42)		
	Xpert MTB/RIF Assay	AFB Microscopy	Xpert MTB/RIF Assay	Culture	AFB Microscopy
TB Positive Cases (*n*)	24	10	39	27	12
Sensitivity	88.9 * (70.8–97.6)	37.0 (19.4–57.6)	92.9 † (80.5–98.5)	64.3 (48.0–78.4)	28.6 ‡ (15.7–44.6)
Specificity	91.8 (86.8–95.3)	98.9 (96.1–99.9)	100 (97.8–100.0)	100 (97.8–100.0)	100 (97.8–100.0)
PPV	61.5 (44.6–76.6)	83.3 (51.6–97.9)	100 (91.0–100.0)	100 (87.2–100.0)	100 (73.5–100.0)
NPV	98.2 (95.06–99.6)	91.4 (86.6–94.9)	98.2 (95.0–99.6)	91.8 (86.8–95.3)	84.8 (79.1–89.5)

* Comparing sensitivities of Xpert assay and AFB microscopy with culture positive cases, *p* < 0.001; † Comparing sensitivities between Xpert assay and culture for CRS, *p* = 0.0047; ‡ Comparing the sensitivities between Xpert assay and AFB microscopy for CRS, *p* < 0.001, CI: confidence interval, PPV: positive predictive value, NPV: negative predictive value.

**Table 4:** AUC of the different diagnostic methods when compared to culture and CRS.

Variables	Compared to Culture (*n* = 27)		Compared to CRS (*n* = 42)		
	Xpert MTB/RIF Assay	AFB Microscopy	Xpert MTB/RIF Assay	Culture	AFB Microscopy
AUC	0.904	0.698	0.964	0.821	0.655
Standard error	0.033	0.048	0.02	0.037	0.036
*p* value	<0.001	<0.001	<0.001	<0.001	<0.001
95% CI *	0.840–0.967	0.640–0.793	0.925–1.000	0.748–0.895	0.548–0.726

* CI: Confidence interval.

## Data Availability

The data presented in this study are available on request from the corresponding author. The data are not publicly available, due to ethical restrictions.
